# Associations of Maternal Retinal Vasculature with Subsequent Fetal Growth and Birth Size

**DOI:** 10.1371/journal.pone.0118250

**Published:** 2015-04-24

**Authors:** Ling-Jun Li, Izzuddin Aris, Lin Lin Su, Mya Thway Tint, Carol Yim-Lui Cheung, M. Kamran Ikram, Peter Gluckman, Keith M. Godfrey, Kok Hian Tan, George Yeo, Fabian Yap, Kenneth Kwek, Seang-Mei Saw, Yap-Seng Chong, Tien-Yin Wong, Yung Seng Lee

**Affiliations:** 1 Singapore Eye Research Institute, Singapore National Eye Centre, Singapore; 2 Department of Pediatrics, Yong Loo Lin School of Medicine, National University of Singapore, Singapore; 3 Department of Obstetrics and Gynecology, Yong Loo Lin School of Medicine, National University of Singapore, Singapore; 4 DUKE-NUS Graduate Medical School, Singapore; 5 Departments of Epidemiology & Ophthalmology, Erasmus Medical Centre, Rotterdam, The Netherlands; 6 Memory Aging & Cognition Centre, National University Health System, Singapore; 7 Singapore Institute for Clinical Sciences, Agency for Science, Technology and Research, Singapore; 8 Medical Research Council Lifecourse Epidemiology Unit and NIHR Southampton, Biomedical Research Centre, University of Southampton, Southampton, United Kingdom; 9 KK Women’s and Children’s Hospital, Singapore; 10 Saw Swee Hock School of Public Health, National University of Singapore, Singapore; 11 Department of Ophthalmology, Yong Loo Lin School of Medicine, National University of Singapore, Singapore; Queen's University, CANADA

## Abstract

**Objective:**

We aimed to study the maternal retinal microvasculature at mid-trimester and its relationship with subsequent fetal growth and birth size.

**Methods:**

We recruited 732 pregnant women aged 18-46 years in the first trimester with singleton pregnancies. All had retinal photography and fetal scan performed at 26-28 weeks gestation, and subsequent fetal scan at 32-34 weeks gestation. Infant anthropometric measurements were done at birth. Retinal microvasculature was measured using computer software from the retinal photographs.

**Results:**

In multiple linear regression models, each 10 μm narrowing in maternal retinal arteriolar caliber was associated with decreases of 1.36 mm in fetal head circumference at 32-34 weeks gestation, as well as decreases of 1.50 mm and 2.30 mm in infant head circumference and birth length at delivery, respectively. Each standard deviation decrease in maternal retinal arteriolar fractal dimension was associated with decreases of 1.55 mm in fetal head circumference at 32-34 weeks gestation, as well as decreases of 1.08 mm and 46.42 g in infant head circumference and birth weight at delivery, respectively.

**Conclusions:**

Narrower retinal arteriolar caliber and a sparser retinal vascular network in mothers, reflecting a suboptimal uteroplacental microvasculature during mid-pregnancy, were associated with poorer fetal growth and birth size.

## Introduction

The placental circulation supports fetal growth, and a reduction in placental blood flow may lead to intrauterine growth restriction (IUGR)[1–6]. Histopathological studies in animal model experiments have shown that smaller placentas are associated with adverse changes in the placental microvasculature—such as decreased capillary volume and capillary area density[7–11] which might indicate reduced placental blood flow. Although the placental and non-placental vasculatures are anatomically and functionally distinct and regulation of perfusion through these vascular beds differs, placental blood flow still approximates 80% to 90% of total uterine blood flow at term pregnancy in primate studies[12]. This indicates that total uterine vascular responses will appear similar to those of the placental vessels[13].

In the past two decades, researchers have found that uterine artery Doppler resistance indexes (RI) at either the second or even as early as the first trimester were associated with subsequent development of abnormal fetal growth, including small-for-gestational-age pregnancy and IUGR[14–19]. Therefore, clinicians treated pregnant women with pre-existing IUGR and pre-eclampsia using pharmacological agents which had a significant effect on reducing uterine artery RI and ultimately improved the utero-placental circulation[20–22]. Nevertheless, abnormalities in this utero-placenta-fetal complex network due to a number of exogenous factors may contribute to poor fetal growth. Even though the current method using ultrasonography assesses utero-placental blood flow supply by measuring uterine artery or/and umbilical artery pulsatility index/resistance index, it does not assess the microvasculature of this complex directly.

The retinal blood vessels of 100–300 μm in size can be visualized non-invasively[23]. Recent epidemiological studies have indicated that retinal vasculature reflects changes in systemic diseases and shares anatomical and physiological similarities with microvasculature in other major organs such as brain, heart, and kidney[24–29]. Thus, it offers an opportunity to study general microvasculature *in vivo* and subsequently monitor microvascular changes which may reflect systemic changes such as elevated blood pressure. Better retinal microvascular signs imply better blood flow not only locally but also systemically. The maternal cardiovascular system undergoes profound changes during pregnancy with hemodynamic adaptation (such as increases in cardiac output and stroke volume) and decreases in peripheral blood pressure and vascular resistance[30–32], which ultimately leads to systemic changes and affects the blood circulation in other organs[33,34]. Hence, we hypothesize that retinal microvasculature might reflect the maternal systemic changes due to pregnancy and especially in the uterine microcirculation during pregnancy, which eventually relates to fetal growth throughout gestation.

Therefore, in the present study, we studied the relationship between maternal retinal microvasculature at mid-term pregnancy, and subsequent fetal growth at later gestation and birth size at delivery.

## Patients and Methods

### Study population

We recruited 1163 women with singleton pregnancies in their first trimester from an on-going birth cohort study, the Growing Up in Singapore Towards Healthy Outcomes (GUSTO) cohort, from June 2009 to Sep 2010. The details of the study are reported elsewhere[35–37]: Briefly, this cohort included Singaporean residents aged 18 years and above, attending the first trimester antenatal clinic at the maternity units of two major government hospital, namely the KK Women's and Children's Hospital (KKH) and the National University Hospital (NUH). The cohort was restricted to those intending to eventually deliver in the above named hospitals and to reside in Singapore for the next 5 years.

Due to the logistic issues, only participants from KKH who were willing to take retinal examination after detailed explanation were further invited to our retinal sub-study. There were no strict exclusion criteria in this retinal sub-study. Overall, 732 out of 952 pregnant subjects with singleton pregnancy without ocular complications had completed retinal photography at 26–28 weeks gestation, fetal scan both at 26–28 weeks and 32–34 weeks gestation, and infant anthropometric measurements at birth. All retinal photographs were gradable.

### Ethics Statement

This study was approved by both SingHealth Centralized Institutional Review Board and the National Health Group’s Domain Specific Review Board, and it was conducted according to the tenets of the Declaration of Helsinki. Written informed consent in 3 copies were obtained from participants prior to any examination. Among these 3 copies, 1 was kept by the mothers, 1 was for the study principal investigator and another was kept at the research site. Singhealth Centralized Institutional Review Board had approved such consent procedure before this study was conduct.

### Retinal Photography and Measurements of Retinal Vascular Parameters

**Fig 1:**
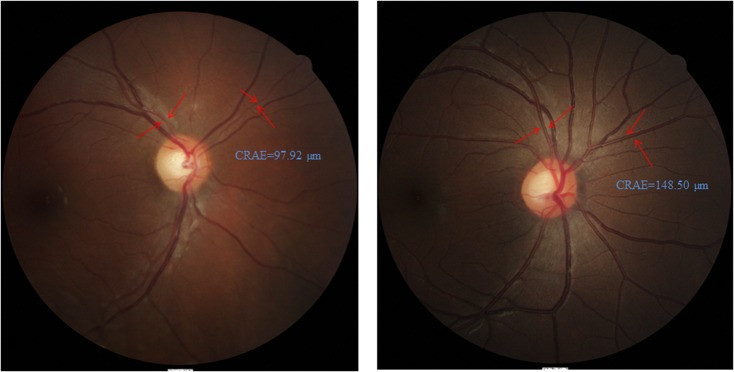
Examples of retinal fundus photographs in our cohort. Central retinal arteriolar equivalent (CRAE) of both images were indicated by the red arrows. The CRAE of Fig 1A and 1B were 97.92 μm and 148.50 μm, respectively. Compared with mother of Fig 1B, mother of Fig 1A showed a narrower CRAE.

**Fig 2:**
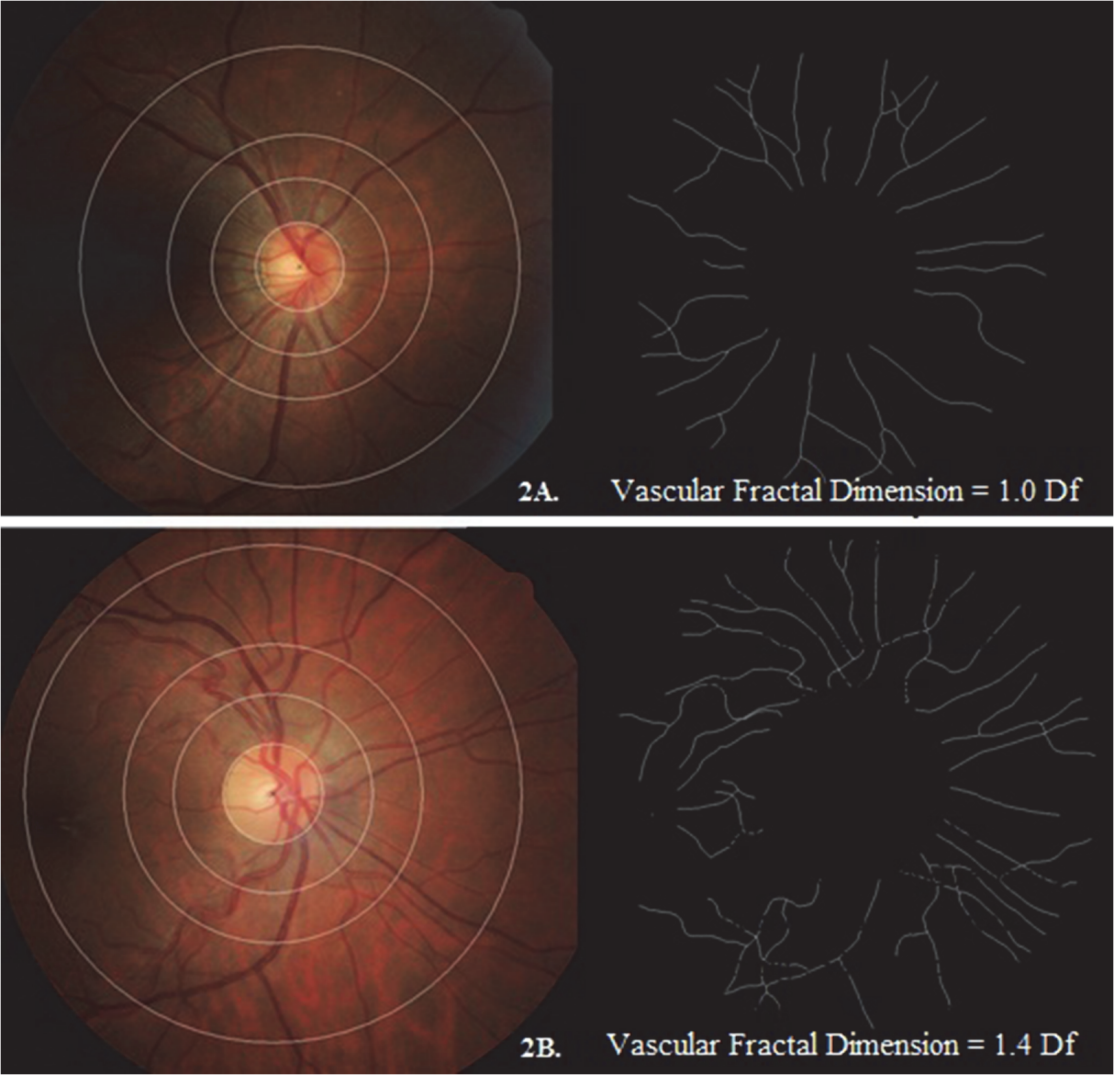
Examples of retinal fundus photographs with relevant pattern of retinal vascular fractal dimension in our cohort. The fractal dimension was shown in black and white images. The retinal vascular fractal dimension of Fig 2A and 2B were 1.0 Df and 1.4 Df, respectively. Compared with mother of Fig 2B, mother of Fig 2A showed a sparser retinal vascular fractal dimension.

Retinal photographs of the right eye were taken without pharmacological pupil dilation using a 45° non-mydriatic retinal camera (Canon CR-1, 40D SLR digital retinal camera backing, Canon Inc, Japan). All retinal photographs were centered on the optic disc and then used for the measurement of retinal vascular parameters according to standardized protocols as described previously in the general population[23,36]. Photographs were assessed by a trained grader using a semi-automated computer-based program (Singapore I Vessel Assessment [SIVA] version 3.0, Singapore Eye Research Institute, Singapore) and the following retinal vascular parameters were assessed:

- Retinal vascular caliber, represented as central retinal arteriolar equivalent (CRAE) and central retinal venular equivalent (CRVE)[23], was assessed. Morphological differences between normal and narrow CRAE among our subjects are shown in **Fig 1**.

- Retinal fractal dimension, which quantifies the complexity of the branching pattern and density of the retinal vessels[23], was also assessed. The fractal dimension is usually a ratio and has no units. The SIVA software calculated the fractal dimension (Df) from the refined skeletonized line tracing using the box-counting method[38–40], an established method used to measure fractal dimension of real life structures that are not perfectly self-similar. Briefly, the box-counting method involves dividing the digital photograph into many squares of a given side length and counting the number of boxes[38–40]. This is repeated for various side lengths and the plot of the logarithms of the number of boxes and the size of the boxes is plotted; its gradient is the measured fractal dimension. Morphological differences between optimal and sparse retinal vascular fractal dimension among our subjects are shown in **Fig 2**.

The gradability of all the mothers’ retinal photographs were up to 98% as reported in previous publications for the same cohort[3,36,37]. Intra-grader reliability was assessed in a sample of 75 (10% of all retinal photographs) randomly selected retinal photographs from the GUSTO cohort. The intra-class correlation coefficients ranged from 0.92–0.97 for retinal vascular parameters.

### Fetal Ultrasonography and Neonatal Anthropometry

Fetal ultrasound scans were performed at 26–28 weeks and 32–34 weeks gestation (model: Aloka SSD-4000, Osaka, Japan). Fetal head circumference, fetal abdominal circumference and femur length were measured based on the standard views (Fetal Medicine Foundation)[41,42]. For measurements at birth, a calibrated infant scale (SECA 334 weighing scale, SECA Corp, Hamburg, Germany) and an infantometer (SECA 210 mobile measuring mat) were used to measure the infant’s weight and recumbent length[43]. Head circumference and abdominal circumference were measured by using a non-stretchable measuring tape (SECA 212 Measuring Tape, SECA Corp.) and subsequently recorded to the nearest 0.1 cm.

Estimated fetal weight (EFW) (gram) at 26–28 and 32–34 weeks gestation was calculated by formula) [44]: Log10 EFW = -1.749 + 0.166 (biparietal diameter [mm]) + 0.046 (abdominal circumference [mm])- 2.646 (abdominal circumference [mm] x biparietal diameter [mm])/100. Estimated fetal length (cm) for 26–28 weeks and 32–34 weeks was calculated by formula) [45]: 6.18 + 0.59 x femur length (mm). In order to facilitate comparison across fetal and infant physical parameters, we converted femur length to estimated fetal length for 3 time points’ comparison for consistency. Since the femur is the longest and most rapidly growing long bone of the fetus, it is a reasonable approach to estimate fetal length on the basis of the femur length[45].

### Other Measurements and Covariates

Maternal upper arm blood pressure was measured using the automatic Omron sphygmomanometer (Omron HEM 705 LP, Omron Healthcare Inc, USA) according to standard protocols[46]. Standing height was measured by SECA model 213 (Seca, Hamburg, Germany) while weight was measured by SECA model 803 (Seca, Hamburg, Germany) according to standard protocols[37]. Body mass index (BMI) was calculated as weight divided by the height squared (kilograms per meter squared). Maternal gestational diabetes mellitus was diagnosed according to WHO guideline (fasting or 2-hr plasma glucose concentrations greater than 7.0 or 7.8 mmol/L respectively) based on 26–28 weeks Oral Glucose Tolerance Test (OGTT) results.

Questions on socio-demographic characteristics (e.g. household income), life style factors (e.g. maternal smoking history), past medical history (e.g. hypertension and diabetes history) and birth order were administered to parents in either English, Chinese, Malay or Tamil during clinical interviews.

### Statistical Analysis

Anthropometric measurements estimated by fetal ultrasonography and measured at birth were all analyzed as continuous variables. Difference in fetal anthropometric measurements between 32–34 weeks and 26–28 weeks, and difference in fetal anthropometric measurements between birth and 26–28 weeks were also analyzed continuously.

Multiple linear regression models were constructed to assess the longitudinal association of baseline maternal retinal vascular parameters at 26–28 weeks gestation and fetal/infant anthropometric measurements at 32–34 weeks and at birth, respectively. In addition, multiple linear regression models were constructed to evaluate the longitudinal association between baseline retinal vascular parameters and fetal growth difference between 32–34 weeks and 26–28 weeks, and between at birth and 26–28 weeks, respectively. Z scores were derived and used to examine the relative growth of individual fetus compared to the fetal growth within the same GUSTO cohort. Therefore, fetal/neonatal growth parameters Z scores were generated at 3 time points: 26–28 weeks, 32–34 weeks, and birth. Mixed model was applied in assessing the association between baseline maternal retinal vascular parameters and fetal growth pattern by using Z score difference in between clinical visits. Stepwise backward methods were selected to choose the most parsimonious and best fitting model. Retinal vascular caliber and fractal dimension were analyzed as independent variables and fetal/neonatal growth parameters were analyzed as dependent variables in 2 models at different time points. Model 1 (32–34 weeks) was adjusted for age, ethnicity, household income, maternal smoking history, birth order, hypertension history, gestational diabetes mellitus diagnosed at 26 weeks, maternal body mass index at 26 weeks, offspring’s gender, and related fetal physical parameters at baseline. Model 2 (at birth) was multivariate-adjusted including variables in model 1 and gestational age at delivery. Furthermore, retinal vascular caliber and fractal dimension were analyzed as independent variables and fetal/neonatal biometry changes were analyzed as dependent variables in 2 models at different time points. Model 3 (Difference between 32–34 and 26–28 weeks) was adjusted for age, ethnicity, household income, maternal smoking history, birth order, hypertension history, gestational diabetes mellitus diagnosed at 26 weeks, maternal body mass index at 26 weeks and offspring’s gender. Model 4 (Difference between at birth and 26–28 weeks) was multivariate-adjusted including variables in model 3 and gestational age at delivery. Fellow vessels were further adjusted in models while retinal arteriolar or venular caliber was involved as independent variables. The reason for adjusting fellow vessel is because that retinal arteriolar caliber and retinal venular caliber account for around 30% of mutual variability due to shared genetic and ocular factors[47].

A significant p value (2-tailed) was defined as <0.05. All statistical analyses were performed using PASW 19.0 (SPSS Inc, Chicago, U.S.).

## Results

**Table 1**shows the baseline characteristics in 3 ethnic groups in our cohort. None of our participants developed preeclampsia during the whole pregnancy and delivery.

**Table 1:** Baseline characteristics across 3 ethnic groups in GUSTO retinal sub-study.

Independent variables	Chinese (n = 398)		Malay (n = 209)		Indian (n = 125)	
	n	Mean (SD) or %	n	Mean (SD) or %	n	Mean (SD) or %
**Age, yrs**	398	31.79 (4.98)	209	28.68 (5.52)	125	30.08 (5.08)
**Household Income**						
SGD ≥6000 per month	117	29.4%	12	5.7%	24	19.2%
**Hypertension history**						
Yes	8	2.0%	9	4.3%	2	1.6%
**Diabetes history**						
Yes	4	1.0%	2	1.0%	2	1.6%
**Gestational diabetes diagnosis (GDM) (at 26–26 wks)**						
Yes	68	18.5%	23	11.7%	25	23.2%
**Smoking history**						
Current or past smokers	36	09.0%	49	23.4%	7	5.6%
**Alcohol drinking history**						
Current or past drinkers	157	39.5%	14	6.7%	16	12.8%
**In-Vitro Fertilization**						
Yes	51	12.8%	6	2.9%	7	5.6%
**Maternal Major Measurement (at 26–28 weeks)**						
Mean arterial pressure, mmHg	315	80.41 (8.83)	173	83.61 (8.72)	97	82.09 (9.88)
Body mass index, kg/m^2^	398	25.14 (3.79)	209	28.07 (5.37)	125	27.68 (4.50)
**Retinal vascular parameters (at 26–28 wks)**						
Arteriolar caliber, μm	398	119.89 (9.03)	209	121.83 (9.21)	125	122.16 (8.88)
Venular caliber, μm	398	169.11 (12.31)	209	175.16 (12.89)	125	171.04 (12.40)
Arteriolar fractal dimension, Df	398	1.25 (0.05)	209	1.26 (0.05)	125	1.26 (0.05)
Venular fractal dimension, Df	398	1.23 (0.05)	209	1.24 (0.05)	125	1.23 (0.04)
**Fetal scan at 26 weeks**						
Head circumference, cm	394	24.93 (1.50)	206	25.02 (1.24)	123	24.86 (1.16)
Abdominal circumference, cm	394	22.15 (1.35)	206	22.27 (1.85)	123	21.66 (1.95)
Estimated birth weight, g	393	1089.81 (182.08)	205	1106.72 (208.17)	122	1051.98 (185.68)
Estimated birth length, cm	393	34.91 (1.71)	206	35.01 (1.78)	123	35.79 (1.76)
**Fetal scan at 32 weeks**						
Head circumference, cm	397	29.84 (1.04)	208	29.98 (1.29)	123	29.67 (1.22)
Abdominal circumference, cm	398	28.15 (1.81)	209	28.41 (1.59)	125	27.83 (1.64)
Estimated weight, g	397	2184.35 (295.26)	208	2224.12 (316.31)	123	2102.40 (33.18)
Estimated length, cm	396	41.88 (1.56)	209	42.24 (1.59)	125	42.90 (1.94)
**Baby measurement at delivery**						
Head circumference, cm	384	33.65 (1.39)	195	33.65 (1.37)	117	33.46 (1.36)
Abdominal circumference, cm	385	28.58 (2.36)	195	28.80 (2.58)	117	28.22 (2.51)
Birth weight, g	383	3096.52 (418.31)	195	3096.02(421.53)	117	3025.82 (400.86)
Birth length, cm	383	48.36 (2.18)	195	48.07 (1.94)	117	48.40 (1.83)

SD, standard deviation.

**Table 2**shows that baseline maternal retinal vasculature at 26–28 weeks was associated with subsequent fetal physical growth—both at 32–34 weeks and at delivery. The results in this table suggested that narrower maternal arteriolar caliber and suboptimal retinal arteriolar fractal dimension at 26–28 weeks were associated with smaller fetal size both at 32–34 weeks and even at birth.

**Table 2:** Multiple linear regression of maternal retinal microvasculature at 26–28 weeks vs. fetal biometry at 32–34 weeks and neonatal biometry at birth.

	Head circumference		Abdominal circumference		Weight		Length	
	β (SE), mm		β (SE), mm		β (SE), g		β (SE), mm	
	32 wks^1^	Delivery^2^	32 wks^1^	Delivery^2^	32 wks^1^	Delivery^2^	32 wks^1^	Delivery^2^
Caliber, per 10 μm ↓ *								
Arteriole	-1.36 (0.60)	-1.50 (0.70)	-1.68 (0.89)	-2.30 (1.40)	-48.26 (14.87)	-28.86 (19.89)	-2.80 (0.80)	-2.30 (1.00)
p value	0.02	0.03	0.06	0.09	0.001	0.15	0.001	0.03
Venule	0.34 (0.43)	1.10 (0.50)	0.42 (0.65)	1.70 (1.00)	20.19 (10.79)	16.91 (14.47)	0.80 (0.60)	1.00 (0.70)
p value	0.43	0.03	0.52	0.08	0.06	0.24	0.18	0.17
**Fractal dimension, per SD ↓**								
Arteriole	-1.55 (0.43)	-1.08 (0.52)	-1.06 (0.67)	-1.44 (0.99)	-27.49 (11.20)	-46.42 (14.68)	-1.88 (0.62)	-1.17 (0.78)
(SD = 0.05Df)								
p value	<0.001	0.04	0.11	0.15	0.01	0.002	0.003	0.13
Venule	-1.10 (0.43)	-0.26 (0.53)	-0.20 (0.68)	-1.41 (1.00)	-16.89 (11.38)	-26.87 (15.02)	-1.36 (0.63)	0.10 (0.79)
(SD = 0.04Df)								
p value	0.01	0.62	0.76	0.16	0.14	0.07	0.03	0.90

SE, standard error; SD, standard deviation.

^1^Model 1: Adjust for age, ethnicity, household income, maternal smoking history, birth order, hypertension history, gestational diabetes mellitus diagnosis at 26 weeks, body mass index at 26 weeks, baby gender and related fetal growth parameters at baseline.

^2^Model 2: Model 1, and gestational age.

*Model for retinal caliber was additionally adjusted for fellow vessel (CRAE for CRVE, CRVE for CRAE).

26 weeks and 32 weeks fetal weight (gram) is calculated by formula (*Shepard et al*): Log 10 EFW = -1.7492 + 0.166 (biparietal diameter [mm]) + 0.046 (abdominal circumference [mm])-2.646 (abdominal circumference [mm] * biparietal diameter [mm])/100

26 weeks and 32 weeks fetal length (cm) is calculated by formula (*Vintzileos et al)*: 6.18 + 0.59 x femur length (mm)

The associations between baseline maternal retinal vasculature at 26–28 weeks and fetal physical growth changes from 26–28 weeks to 32–34 weeks and then eventually to birth are shown in **Table 3**. There were consistent findings showing associations between smaller maternal retinal arteriolar fractal dimension and smaller fetal weight and length growth from 26–28 weeks to delivery. These findings suggested that suboptimal retinal blood flow, as reflected in retinal arteriolar narrowing and smaller retinal arteriolar fractal dimension, at 26–28 weeks gestation was associated with slower fetal growth—both at 32–34 weeks and at delivery.

**Table 3:** Multiple linear regression of maternal retinal microvasculature at 26–28 weeks vs. fetal biometry changes between 32–34 weeks and 26–28 weeks and neonatal biometry changes between delivery and 26–28 weeks.

	Head circumference		Abdominal circumference		Weight		Length	
	β (SE), mm		β (SE), mm		β (SE), g		β (SE), mm	
	Diff between	Diff between	Diff between	Diff between	Diff between	Diff between	Diff between	Diff between
	32–26 week^3^	del-26 week^4^	32–26 week^3^	del-26 week^4^	32–26 week^3^	del-26 week^4^	32–26 week^3^	del-26 week^4^
Caliber, per 10 μm ↓ *								
Arteriole	-1.10 (0.80)	-1.70 (0.90)	-1.40 (1.00)	-2.50 (1.60)	-47.75 (15.05)	-54.20 (23.11)	-2.40 (1.00)	-3.00 (1.30)
p value	0.19	0.06	0.16	0.11	0.002	0.02	0.02	0.02
Venule	-0.40 (0.60)	0.40 (0.70)	-0.30 (0.70)	0.60 (1.10)	16.77 (10.89)	13.48 (16.86)	0.00 (0.70)	0.200 (1.00)
p value	0.50	0.51	0.71	0.57	0.12	0.42	0.99	0.82
**Fractal dimension, per SD ↓**								
Arteriole	-1.09 (0.61)	-0.49 (0.67)	-0.94 (0.75)	-1.05 (1.10)	-26.09 (11.36)	-42.13 (15.10)	-1.58 (0.76)	-0.67 (0.93)
(SD = 0.05Df)								
p value	0.08	0.46	0.21	0.34	0.02	0.003	0.04	0.47
Venule	-1.03 (0.62)	-0.12 (0.68)	-0.43 (0.76)	-1.56 (1.12)	-18.03 (11.53)	-27.64 (15.44)	-1.26 (0.76)	0.36 (0.94)
(SD = 0.04Df)								
p value	0.10	0.86	0.57	0.16	0.12	0.07	0.10	0.70

SE, standard error; SD, standard deviation.

^3^Model 3: Adjust for age, ethnicity, household income, maternal smoking history, birth order, hypertension history, gestational diabetes mellitus diagnosis at 26 weeks, body mass index at 26 weeks and baby gender.

^4^Model 4: Model 1 and gestational age.

*Model for retinal caliber was additionally adjusted for fellow vessel (CRAE for CRVE, CRVE for CRAE).

A mixed model was applied to study the association between maternal retinal vascular parameters at baseline and fetal growth pattern based on repeated measurements at 3 time points. **Table 4**showed the association between maternal retinal vascular parameters in tertiles and standard deviation in fetal/neonatal weight and length changes across 3 time points at 26–28 weeks, at 32–34 weeks and at delivery. Mixed model showed that mothers’ retinal vascular parameters in the lowest values (1^st^ tertile) had the slowest fetal weight gain according to the Z score compared with mothers’ retinal vascular parameters in higher values.

**Table 4:** Mixed models of associations between maternal retinal vascular parameters in tertiles and standard deviation in fetal/neonatal weight and length changes across 3 time points at 26–28 weeks, at 32–34 weeks and at delivery.

	SD in weight			SD in length		
	mean difference (SE)	p^1^	p^2^	mean difference (SE)	p^1^	p^2^
**CRAE**						
1^st^ vs. 2^nd^ tertile	-0.13 (0.09)	0.16	0.04	-0.14 (0.09)	0.14	0.02
1^st^ vs. 3^rd^ tertile	-0.23 (0.09)	0.01		-0.26 (1.00)	0.002	
**CRVE**						
1^st^ vs. 2^nd^ tertile	0.05 (0.10)	0.61	0.87	0.07 (0.10)	0.45	0.64
1^st^ vs. 3^rd^ tertile	0.03 (0.09)	0.72		0.08 (0.10)	0.38	
**Retinal arteriolar fractal dimension**						
1^st^ vs. 2^nd^ tertile	0.01 (0.09)	0.90	0.67	0.17 (0.09)	0.21	0.20
1^st^ vs. 3^rd^ tertile	-0.07 (0.09)	0.49		-0.05 (0.10)	0.64	
**Retinal venular fractal dimension**						
1^st^ vs. 2^nd^ tertile	-0.21 (0.09)	0.02	0.07	-0.15 (0.09)	0.11	0.26
1^st^ vs. 3^rd^ tertile	-0.13 (0.09)	0.17		-0.04 (0.10)	0.66	

SD, standard deviation; SE, stand error; CRAE, central retinal arteriolar equivalent; CRVE, central retinal venular equivalent.

^1^ for pairwise comparison

^2^ F test tests the effect of retinal vascular parameter-tertile trend.

No effect modifier was found in our multivariate analysis.

## Discussion

In this multi-ethnic cohort study, we found that suboptimal microvasculature reflected by smaller retinal arteriolar caliber and sparser vascular fractal dimension at mid-term pregnancy was associated with poorer fetal growth and birth size.

The human placenta is a complex villous structure that greatly increases the contact surface area between the mother’s blood space and the fetal circulation[32,48]. Without an increase in maternal blood flow, preterm birth and fetal loss will occur[6]. In human beings, evolution has selected the mechanism of increasing maternal blood flow to placental bed through the uterine spiral arteries[19]. It is well known that changes in placental transport capacity and perfusion might lead to poorer placental circulation which results in IUGR[7–10]. Several experimental approaches in animals have been developed to assess placental perfusion such as radioactive microsphere and angiography[49], and even magnetic resonance imaging (MRI) and superparamagnetic iron oxide (SPIO)[1]. In human pregnancy, the conventional way to monitor the occurrence of impaired utero-placental blood flow and intrauterine growth retardation is to perform Doppler ultrasonography to assess blood flow velocity and measure fetal biometry[1]. For example, clinical studies have been using uterine artery resistance indexes (RI) as a surrogate for placental perfusion, and indirectly linked uterine artery RI to fetal outcomes such as small-for-gestational-age pregnancy and IUGR[14–19]. However, ultrasonography has questionable reliability due to the subjective intra-/inter-grader measurements[50–53]. Furthermore, subclinical morphological differences in the unit of small vessels that lead to abnormal fetal growth might not be detected by ultrasonography. There is a growing need in assessing the utero-placental microvasculature objectively, repeatedly and non-invasively.

Clinical research has studied skin microvascular function using acetylcholine response on pregnant women, and it showed a positive correlation between birth weight and endothelial function (r = 0.317, p = 0.022) at 22 weeks gestation[3]. Researchers from this study suggested that during normal pregnancy changes in micro-vascular function might reflect important adaptations that were required to facilitate normal fetal growth. In addition, another study showed that there was a clear difference in vascular ultra-structure of the placentas of pregnancies with IUGR compared with controls with normal pregnancy [54]. Such morphological abnormalities included decreased branching of arterioles and venules, increased total amount of blood vessels, more dense and aberrant blood vessels with a very tortuous course in capillary network. All these pathological changes in placental microvascular structure implied impedance in utero-placental blood flow during gestation in the cause of IUGR[54–56].

The retinal vascular tree and its branching pattern capture the “optimal state” of the retinal microvasculature. In the last decade, retinal microvasculature has been used as a surrogate for systemic microcirculation. Theoretical and experimental work supports the concept that the vascular architecture develops in a way of optimizing the efficient flow, and any deviation from this optimal state indicates a certain disease process[57]. There are a series of parameters representing the retinal microvasculature, and overall they reflect the optimacy of the retinal blood flow, such as caliber and fractal dimension[24,36,37,58,59]. Fractal analysis is a method to quantify the geometric branching complexity and density of the retinal vessels. A fractal is a type of geometric pattern that permits the characterization of objects that branch repeatedly, such as the blood vessels in the heart and lungs. It can be summarized by the fractal dimension, which measures the complexity of the branching pattern. Thus, a “global” measure summarizing the whole branching pattern of the retinal vascular tree as a single parameter would clearly be useful. For example, lower fractal dimension in retinal vessels indicates rarefaction or loss of vessels, whereas higher fractal dimension indicates a more complex retinal vascular network or a microvascular proliferation[60]. In summary, these observations of retinal vascular caliber and fractal dimension show that vascular architecture develops in a way that is optimized for efficient flow, and that deviations from this optimal state occur in disease processes[61].

Retinal vascular caliber and fractal dimension have been shown to be the most consistent retinal parameters which are associated with cardiovascular risks like obesity, hyperlipidemia, and hyperglycemia, and systemic diseases like hypertension and diabetes [24,59,60]. During pregnancy, a woman’s total blood volume increase by about 40% and her cardiac output rises by about 30–35%[30]. Since maternal cardiovascular system undergoes profound changes during pregnancy with hemodynamic adaptation[33,34], it ultimately leads to systemic changes and increment of blood circulation in major organs such as uteroplacental blood flow accounting for 25% of a woman’s cardiac output[30–32]. The novelty of our study is to use retinal imaging to assess maternal microvasculature *in vivo* as early as mid-term pregnancy, which might provide additional and valuable information about intrauterine fetal growth mediated by uteroplacental perfusion. Our previous studies have shown that a series of morphological abnormalities on retinal vascular caliber and fractal dimension among the same GUSTO pregnant women were associated with maternal systemic factors (e.g. elevated blood pressure and obesity)[36,37].

Our study suggests a role of the maternal retinal microvasculature in association with subsequent fetal growth and birth size. The findings showed that smaller retinal arteriolar caliber and sparser vascular network among pregnant women at 26–28 weeks gestation was associated with a certain amount of decrement in fetal growth parameters such as head circumference, estimated fetal weight and length in late pregnancy and even birth weight and length until delivery. Retinal vascular morphologic differences may be a sign of suboptimal blood flow in retinal microcirculation, and also may reflect suboptimal maternal microcirculation elsewhere, including the uterine bed and placenta. Our findings suggested that during pregnancy, suboptimal retinal vasculature at 26–28 weeks gestation was associated with a smaller fetal development both at 32–34 weeks gestation and birth size.

The main strengths of our study are longitudinal design and standardized procedures. The longitudinal design of our study suggested that changes in the circulatory system reflected by the retinal vessels have a causal relationship with fetal growth. Furthermore, this study was conducted by following standardized protocols, using validated assessments of retinal vasculature, and achieving detailed information on a range of potential confounders. However, there were few methodological issues that are limitations of this study. Firstly, there might be a limited role for selection bias in the 732 participants selected from a total number of 952 eligible subjects, even though we did not find any significant differences in baseline characteristics between these 2 groups. Secondly, information bias (e.g. smoking and alcohol drinking) might occur on confounders which were mainly collected during subjective questionnaire interviews.

In summary, in this cohort on 732 pregnant women, we showed that narrower retinal arteriolar caliber and a sparser vascular network, indicative of suboptimal retinal microvasculature, is associated with poorer fetal growth in late pregnancy and poorer birth size at birth.
